# Supplementary material for: Titanate Nanotubes Engineered with Gold Nanoparticles and Docetaxel to Enhance Radiotherapy on Xenografted Prostate Tumors
Source: Cancers (Basel). 2019 Dec 6;11(12):1962. doi: 10.3390/cancers11121962 (PMC6966691; doi:10.3390/cancers11121962)
Supplement: Supplementary file 1 [file cancers-11-01962-s001.pdf]

Supplementary Materials

Titanate Nanotubes Engineered with Gold Nanoparticles and Docetaxel to Enhance Radiotherapy on Xenografted Prostate Tumors

Alexis Loiseau[1], Julien Boudon, Alexandra Oudot, Mathieu Moreau, Romain Boidot, Rémi Chassagnon, Nasser Mohamed Saïd, Stéphane Roux, Céline Mirjolet and Nadine Millot

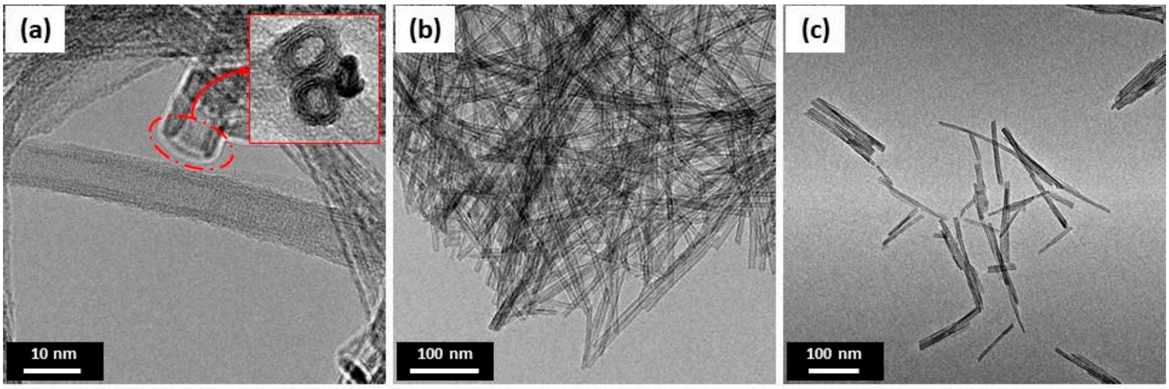

Figure S1. TEM images show (**a**) the spiral morphology with an internal cavity of titanate nanotubes and (**b,c**) the evolution of TiONts dispersion before and after APTES grafting, respectively.

$$N_{OH} = \frac{2 \times \frac{\Delta m}{m} \times \mathcal{N}_A}{\mathrm{M(H_2O)} \times \mathrm{S_{BET}} \times 10^{18}} \quad (1)$$

The ratio for the hydroxyl group at the surface of bare TiONts was calculated from this equation knowing that the condensation of one water molecule involves two hydroxyls. This weight loss correlated with chemisorbed water between 190 and 800 °C, while the first loss was mainly due to physisorbed water.

$\mathrm{N_{OH}}$ is the number of the hydroxyl groups per nm^2, Δm is the relative mass loss of chemisorbed water determined by derivative curve (g), m is the initial sample mass (g), $\mathcal{N}_A$ is Avogadro's constant, $\mathrm{M_{H_2O}}$ is the molecular weight of water (g.mol^{-1}), $\mathrm{S_{BET}}$ is the specific surface area (m^2.g^{-1}) and 10^{18} is a factor to obtain a ratio per nm^2.

Figure S2. Theoretical calculation of the hydroxyl rates on bare TiONts.

$$N_m = \frac{\left(\frac{\Delta m_2}{m_2} - \frac{\Delta m_1}{m_1}\right) \times \mathcal{N}_A}{\mathrm{M}(m_2) \times \mathrm{S_{BET}} \times 10^{18}} \quad (2)$$

Mass losses increased as additional organic moieties were added to TiONts at each successive step of grafting. $\mathrm{N_m}$ is the number of grafted m molecules per nm^2, $\left(\frac{\Delta m_2}{m_2} - \frac{\Delta m_1}{m_1}\right)$ is the difference in relative mass loss between two samples, $\mathcal{N}_A$ is Avogadro's constant, $\mathrm{M}(m_2)$ is the molecular weight of the degraded molecule (g.mol^{-1}), $\mathrm{S_{BET}}$ is the specific surface area (m^2.g^{-1}) and 10^{18} is a factor to obtain a ratio per nm^2.

Figure S3. Theoretical calculation for functionalized-TiONts rate of grafting.

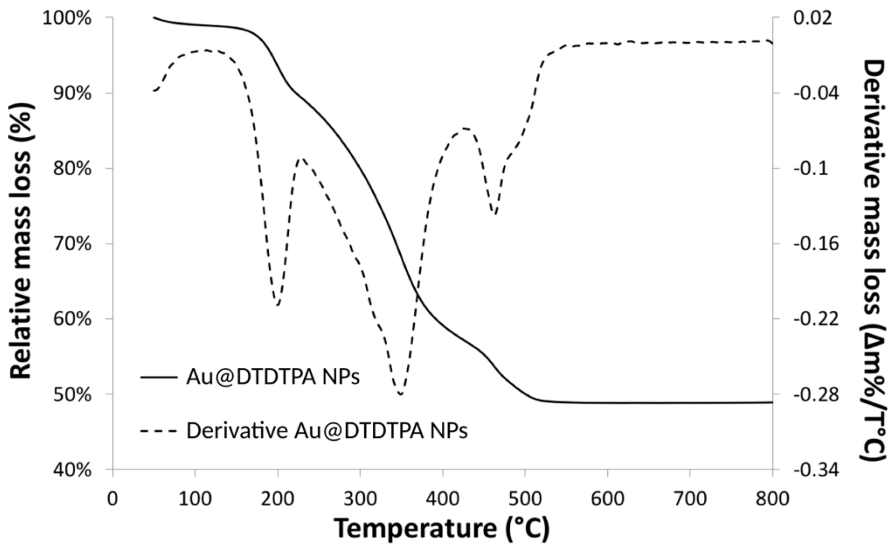

Nanohybrid Name	Initial Temperature of Degradation (°C)	Relative Mass Loss (%)	Degraded Molecular Weight (g.mol^{-1})	Molecule.nm^{-2} (average)	Number of Grafted Molecules per AuNP [1]
Au@DTDTPA NPs	105	50.2	511	5.7 DTDTPA	120

Figure S4. TGA and derivative curves of Au@DTDTPA NPs under air atmosphere. The results of relative mass loss and graft ratio of Au@DTDTPA NPs are summarized (S_{BET} = 104 m^2.g^{-1}). [1] The number of grafted molecules per AuNP estimated by means of geometrical calculation considering only the external surface of AuNPs.

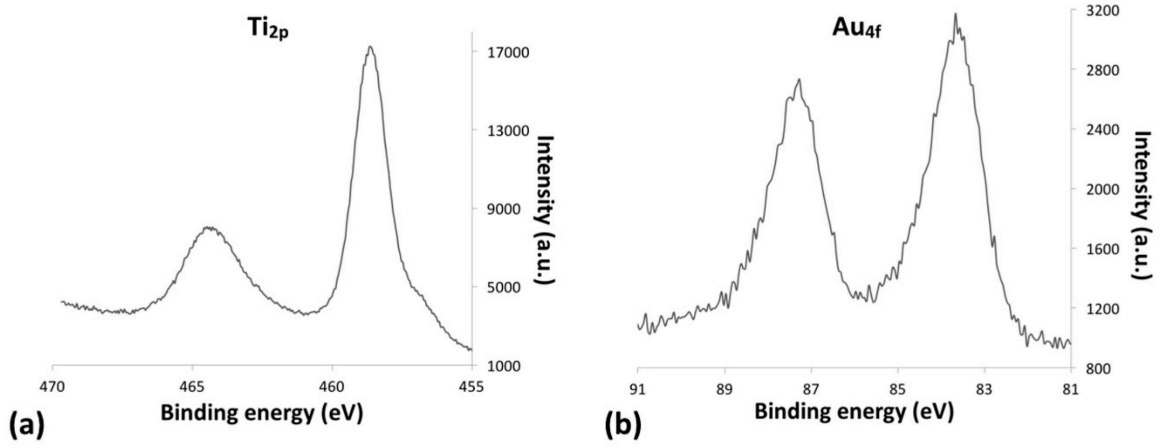

Figure S5. (**a**) Ti_{2p} and (**b**) Au_{4f} peaks in XPS spectra for TiONts-AuNPs.

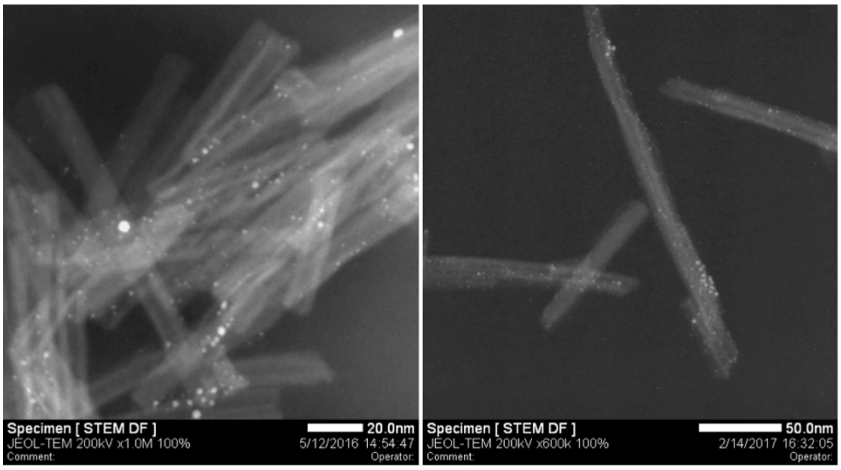

Figure S6. HAADF-STEM images of TiONts-AuNPs-PEG3000.

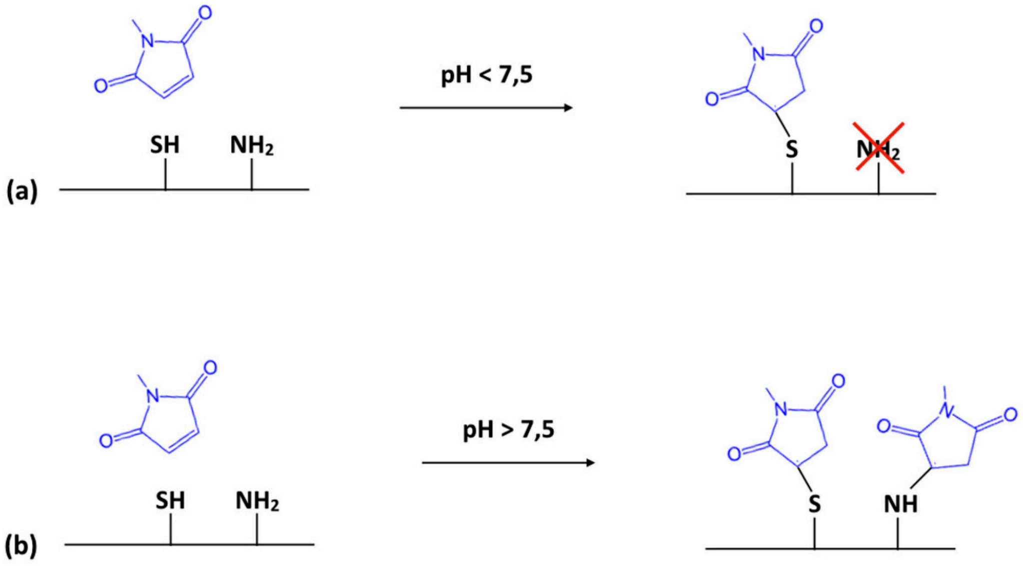

Figure S7. (**a**) Maleimide reacts specifically with a thiol function at pH < 7.5 and (**b**) lose its specificity to react either with a thiol function or with amine function at pH > 7.5.

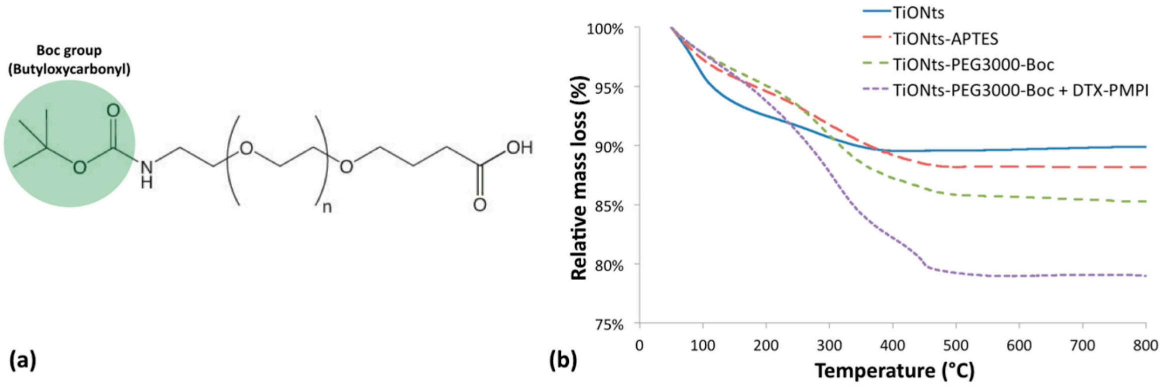

Nanohybrid Name	Initial Temperature of Degradation (°C)	Relative Mass Loss (%)	Degraded Molecular Weight (g.mol^{-1})	Molecule.nm^{-2} (average)	Reproducibility (n)	Number of Grafted Molecules per TiONt (*)
TiONts	190	2.6	18	10.6 (±1.5) OH	10	–
TiONts-APTES	175	6.3	58	5.3 (±0.5) NH_2	7	14,230
TiONts-PEG3000-Boc	165	13.2	3,173	0.060 (±0.003) PEG3000-Boc	2	330
TiONts-PEG3000-Boc + DTX-PMPI	150	17.9	1,022	0.13 (±0.01) DTX-PMPI	2	710

Figure S8. (**a**) Polymer (Boc-NH-PEG3000-COOH; M = 3173 g.mol^{-1}) having an inactive function (Boc) and carboxyl function to react with an amine group *via* peptide coupling and (**b**) TGA curves showing the adsorption of DTX-PMPI upon contact between TiONts-PEG3000-Boc and DTX-PMPI (TiONts-DTX were washed by dialysis and ultrafiltration (100 kDa)).

TGA curves confirmed the presence of modified-DTX on simple contact with an inactive polymer grafted onto TiONts-APTES bearing a Boc function (the presence of which prevented any chemical reaction with DTX; PEG MW = 3,000 g.mol^{-1}, ref. PEG1073), instead of a thiol function (in case of grafting). This observation suggests that there was another interaction mode between

DTX-PMPI and TiONt-AuNPs-PEG_{3000} despite repeated purifications. Consequently, it cannot be excluded that DTX-PMPI clung/adsorbed to amine groups of APTES unfunctionalized by Au@DTDTPA NPs, in the cavity of nanotubes and/or be trapped within PEG chains.

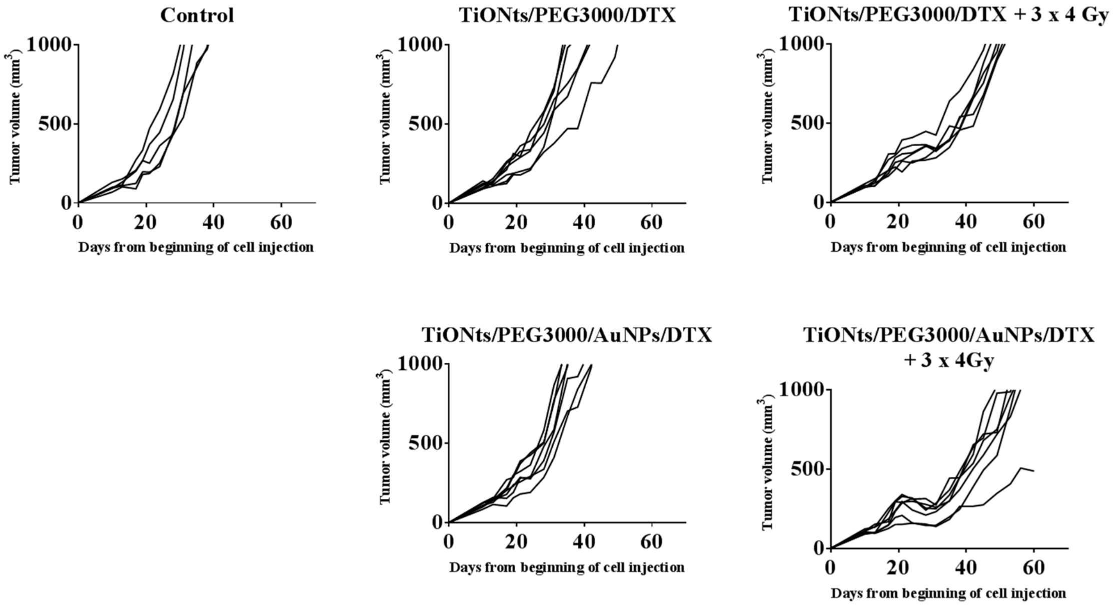

Figure S9. Therapeutic effect as function of time of control, TiONts-PEG_{3000}-DTX and TiONts-AuNPs-PEG_{3000}-DTX, associated or not with radiotherapy (RT) with three daily fractions of 4 Gy, 24 h after injection into PC-3 xenografted tumors (n = 6–7).

© 2019 by the authors. Licensee MDPI, Basel, Switzerland. This article is an open access article distributed under the terms and conditions of the Creative Commons Attribution (CC BY) license (http://creativecommons.org/licenses/by/4.0/).
